# Conformational Dynamics of the D53−D3−D14 Complex in Strigolactone Signaling

**DOI:** 10.1093/pcp/pcad067

**Published:** 2023-06-29

**Authors:** Simiao Liu, Jia Wang, Bin Song, Xinqi Gong, Huihui Liu, Qingliang Hu, Junhui Zhang, Qianqian Li, Jie Zheng, Hongwei Wang, H Eric Xu, Jiayang Li, Bing Wang

**Affiliations:** Beijing Advanced Innovation Center for Structural Biology, Tsinghua-Peking Joint Center for Life Sciences, Center for Plant Biology, School of Life Sciences, Tsinghua University, Beijing 100084, China; The Drug Research Center of Immunological Diseases, Shanghai Institute of Materia Medica, Chinese Academy of Sciences, Shanghai 201203, China; Institute for Mathematical Sciences, Renmin University of China, Beijing 100872, China; State Key Laboratory of Plant Genomics and National Center for Plant Gene Research (Beijing), Institute of Genetics and Developmental Biology, Chinese Academy of Sciences, Beijing 100101, China; State Key Laboratory of Plant Genomics and National Center for Plant Gene Research (Beijing), Institute of Genetics and Developmental Biology, Chinese Academy of Sciences, Beijing 100101, China; State Key Laboratory of Plant Genomics and National Center for Plant Gene Research (Beijing), Institute of Genetics and Developmental Biology, Chinese Academy of Sciences, Beijing 100101, China; University of Chinese Academy of Sciences, Beijing 100049, China; State Key Laboratory of Plant Genomics and National Center for Plant Gene Research (Beijing), Institute of Genetics and Developmental Biology, Chinese Academy of Sciences, Beijing 100101, China; The Drug Research Center of Immunological Diseases, Shanghai Institute of Materia Medica, Chinese Academy of Sciences, Shanghai 201203, China; Beijing Advanced Innovation Center for Structural Biology, Tsinghua-Peking Joint Center for Life Sciences, Center for Plant Biology, School of Life Sciences, Tsinghua University, Beijing 100084, China; State Key Laboratory of Plant Genomics and National Center for Plant Gene Research (Beijing), Institute of Genetics and Developmental Biology, Chinese Academy of Sciences, Beijing 100101, China; The CAS Key Laboratory of Receptor Research and the State Key Laboratory of Drug Research, Shanghai Institute of Materia Medica, Chinese Academy of Sciences, Shanghai 201203, China; State Key Laboratory of Plant Genomics and National Center for Plant Gene Research (Beijing), Institute of Genetics and Developmental Biology, Chinese Academy of Sciences, Beijing 100101, China; University of Chinese Academy of Sciences, Beijing 100049, China; Yazhouwan National Laboratory, Sanya 572025, China; State Key Laboratory of Plant Genomics and National Center for Plant Gene Research (Beijing), Institute of Genetics and Developmental Biology, Chinese Academy of Sciences, Beijing 100101, China; University of Chinese Academy of Sciences, Beijing 100049, China

**Keywords:** D3, D14, D53, Receptor complex, Signal transduction, Strigolactone

## Abstract

Strigolactones (SLs) play fundamental roles in regulating plant architecture, which is a major factor determining crop yield. The perception and signal transduction of SLs require the formation of a complex containing the receptor DWARF14 (D14), an F-box protein D3 and a transcriptional regulator D53 in an SL-dependent manner. Structural and biochemical analyses of D14 and its orthologs DAD2 and AtD14, D3 and the complexes of ASK1−D3−AtD14 and D3^CTH^–D14 have made great contributions to understanding the mechanisms of SL perception. However, structural analyses of D53 and the D53−D3−D14 holo-complex are challenging, and the biochemical mechanism underlying the complex assembly remains poorly understood. Here, we found that apo-D53 was rather flexible and reconstituted the holo-complex containing D53, S-phase kinase-associated protein 1 (SKP1), D3 and D14 with *rac-*GR24. The cryo-electron microscopy (cryo-EM) structure of SKP1−D3−D14 in the presence of D53 was analyzed and superimposed on the crystal structure of ASK1−D3−AtD14 without D53. No large conformational rearrangement was observed, but a 9Å rotation appeared between D14 and AtD14. Using hydrogen–deuterium exchange monitored by mass spectrometry, we analyzed dynamic motifs of D14, D3 and D53 in the D53−SKP1−D3−D14 complex assembly process and further identified two potential interfaces in D53 that are located in the N and D2 domains, respectively. Together, our results uncovered the dynamic conformational changes and built a model of the holo-complex D53−SKP1−D3−D14, offering valuable information for the biochemical and genetic mechanisms of SL perception and signal transduction.

## Introduction

Plant hormones are signaling compounds that regulate growth, development and environmental responses. Strigolactones (SLs) are a class of plant hormones that regulate a variety of growth and developmental processes, such as shoot branching, plant height, leaf morphology, culm strength and root development (Gomez-Roldan et al. 2008, Umehara et al. 2008, Al-Babili and Bouwmeester 2015, Yano et al. 2015, Mashiguchi et al. 2021). SLs also mediate symbiosis and parasitism relationships in the rhizosphere and play important roles in the adaptation of plants to low phosphorus and drought environments (Cook et al. 1966, Conn et al. 2015, Waters et al. 2017, Kyozuka et al. 2022, Wang et al. 2022). As a kind of sesquiterpene lactone, natural SLs typically comprise a variable tricyclic lactone (the ABC rings) that is connected with a conserved butenolide ring (D ring) through an enol–ether bridge, and the separated ABC or D ring is inactive in plants (Zwanenburg et al. 2009, Akiyama et al. 2010, Xie et al. 2013).

In SL perception and signal transduction processes, three highly conserved components have been identified, including rice DWARF14 (D14), D3, D53 and their homologs in other higher plants (Ishikawa et al. 2005, Umehara et al. 2008, Arite et al. 2009, Jiang et al. 2013, Zhou et al. 2013). D14, a member of the α/β serine hydrolase superfamily, functions as the SL receptor and hydrolyzes SLs into tricyclic ABC- and D-ring products (Hamiaux et al. 2012, Zhao et al. 2013, Yao et al. 2016). D3 is an F-box protein that binds S-phase kinase-associated protein 1 (SKP1) in the SKP1–CUL1–F-box (SCF) complex and can recognize its substrate D53 (Stirnberg et al. 2007, Zheng and Shabek 2017). D53 has weak similarity with AAA+ ATPase family proteins and contains three conserved ethylene-responsive element-binding factor–associated amphiphilic repression (EAR) motifs that are required for transcriptional repression activity (Jiang et al. 2013, Zhou et al. 2013). When the SL level is low, D53 interacts with the transcriptional repressor TOPLESS through its EAR motif and inhibits downstream gene expression. With increasing SL levels, D14 binds SLs and forms the D14–D3–D53 complex, which triggers rapid degradation of D53 and subsequent transcriptional and non-transcriptional events (Soundappan et al. 2015, Wang et al. 2015, Liang et al. 2016). Using the SL analog GR24^4DO^, hundreds of SL-responsive genes have been identified in *Arabidopsis*, and SMXL6 was found to be a novel dual-functional repressor protein that can directly interact with DNA or interact with transcription factors to regulate gene expression (Wang et al. 2020a).

To illuminate the recognition mechanism of SLs, structures of apo-D14 and SL-bound forms were solved, and only weak conformational changes were observed (Kagiyama et al. 2013, Nakamura et al. 2013, Zhao et al. 2013, 2015). When ASK1 and D3 were incubated with AtD14 in the presence of SLs, an ASK1–D3–AtD14 complex was formed and showed allosteric activation compared with the apo-state of AtD14. The structure of the ASK1–D3–AtD14 complex showed a hormone hydrolysis state with covalently bound covalently linked intermediate molecule (CLIM) in the His–Ser–Asp catalytic center of D14, indicating that AtD14 had dual functions to generate and sense the active form of SLs (Yao et al. 2016). Another crystal structure of rice SKP1–D3 illuminated that D3 adopted engaged or dislodged states due to conformational changes in the C-terminal helix (CTH). The structure of D14–D3^CTH^ complex showed that the conformation of D14 in complex with D3^CTH^ is consistent with that of apo-D14, suggesting that D14 adopts an open conformation to initiate signal transduction (Shabek et al. 2018). The perception and hydrolysis mechanism of SLs has been a subject of debate. Genetic analyses indicated that an AtD14^D218A^-mutated protein lacking enzymatic activity is still able to complement the *d14* phenotype in an SL-dependent manner, suggesting that the intact SL molecules could trigger SL signaling without hydrolysis. D14 was proposed to be a dual-functional receptor that is responsible for both the perception and deactivation of bioactive SLs (Seto et al. 2019). A recent study showed that the conformational switch in ASK1–D3^CTH^ directly regulated ubiquitin-mediated degradation of D53, and the dislodged conformation was required for SL-triggered D53 recruitment and ubiquitination, while the engaged conformation was required for the release of polyubiquitinated D53 and subsequent degradation of D14 (Tal et al. 2022).

Although multiple modes of the D3–D14 complex have been found in vitro, the structure of D53 has not been solved. The association of D14–D3 with D53 upon SL perception is a key step in SL signaling, and the holo-complex structure of D14–SL–D3–D53 needs to be determined. Here, we analyzed the biochemical characteristics of D53, reported the cryo-EM structure of SKP1–D3–D14 in the presence of D53 and elucidated the conformational dynamics of SKP1–D3, D14 and D53 during SL perception. Our findings provide insights for structural and functional analyses of the D14–SL–SKP1–D3–D53 complex in SL signaling.

## Results

### Reconstitution of a GR24-induced D53–SKP1–D3–D14 complex in vitro

**Fig. 1:**
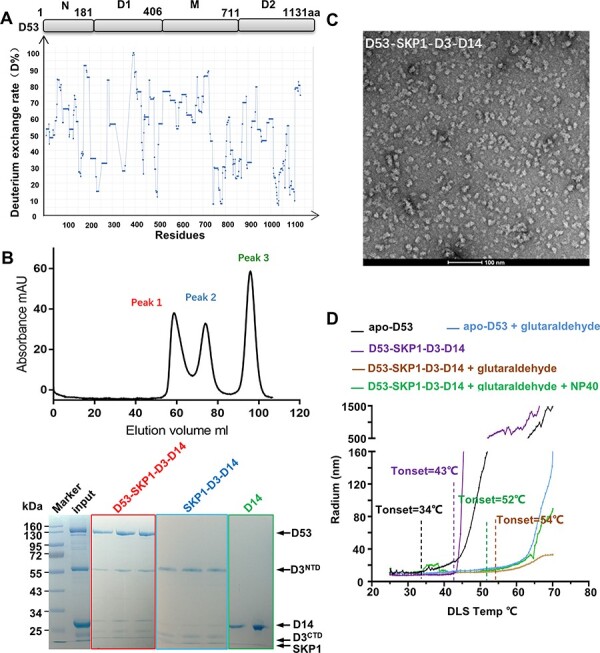
The reconstitution of complex D53−SKP1−D3−D14. (A) The domain composition of D53 with domain boundaries indicated (upper panel) and HDX dynamics of D53 (lower panel). D% represents the rate of hydrogen to deuterium exchange in residues of D53. (B) The size-exclusion chromatography analysis of complex formation among full-length D53, SKP1−D3 and D14 in the presence of *rac*-GR24 (upper panel) and Coomassie blue staining of eluted fractions colored as indicated (peak 1: D53−SKP1−D3−D14; peak 2: SKP1−D3−D14 and peak 3: D14) following SDS−PAGE analysis (lower panel). (C) The representative negative staining image of the D53−SKP1−D3−D14 complex. The scale bar is 100 nm. (D) The thermostability analysis of D53 and D53−SKP1−D3−D14 complexes by DLS. The hydrodynamic radii of the corresponding D53 complexes (colored as indicated) were monitored as a function of temperature.

D53 belongs to the AAA+ ATPase family and comprises four distinct domains: the N-terminal region, the D1 ATPase domain, the bridge M domain and the D2 ATPase domain (**Fig. 1A**). To obtain D53 protein with high purity, full-length D53 protein was expressed in insect cells and subsequently purified by anion exchange and gel-filtration chromatography to homogeneity (**Supplementary Fig. S1A**). The apo-D53 protein was subjected to negative staining and cryo-electron microscopy (cryo-EM) analyses, but D53 particles in both analyses displayed unclear features (**Supplementary Fig. S1B, C**), which may be due to the characteristics of D53. To evaluate the stability of D53, we performed hydrogen–deuterium exchange coupled with mass spectrometry (HDX−MS), which was used to measure changes in protein structure and conformation by monitoring the exchange rates of amide hydrogens on the peptide backbone and deuterium in the solvent (Pascal et al. 2012). We obtained 91.9%, 97.4% and 92.4% peptide coverage for D14, D3 and D53, respectively, allowing us to interrogate the dynamics of entire proteins (**Supplementary Fig. S2**). HDX–MS analysis indicated that 76% amino acids of D53 underwent a high ratio of hydrogen−deuterium exchange (HDX perturbation key, D% ≥ 40%), while the D% of D14 and D3 was approximately 10% in their apo-state (**Fig. 1A**, **Supplementary Fig. S2**), suggesting that more flexible loops and secondary structures of D53 were exposed to solvent. The highly dynamic characteristics of D53 probably led to the disordered particles in negative staining and cryo-EM assays. To improve the stability of D53, we performed thermal shift assays (TSAs) and measured the effects of different buffer conditions containing various bivalent cations. TSA curves of D53 showed two distinct transition peaks, which are often observed in a multidomain protein or a multisubunit complex (Niesen et al. 2007). Compared with the standard buffer condition, the addition of different bivalent cations showed no significant increase in Tm values, indicating that they did not improve the thermostability of D53 (**Supplementary Fig. S1D**). In addition, although crosslinking with glutaraldehyde changed the migration velocity of D53 in the native-gel assay, the particle feature of D53 in the negative staining assay was unimproved (**Supplementary Fig. S1E, F**).

To overcome the barrier of D53 and illustrate the complete SL perception mechanism, we attempted to reconstitute the SL signal perception complex D53−SKP1−D3−D14 in vitro. SKP1 and D3 were coexpressed in insect cells, purified through ion-exchange chromatography and then digested by tobacco etch virus (TEV) protease to obtain an SKP1−D3^NTD/CTD^ complex following a previous report (Shabek et al. 2018). The D3^NTD^ and D3^CTD^ segments in the complex were tightly associated with each other and termed D3 hereafter (**Supplementary Fig. S3A, B**). D14 was overexpressed in *Escherichia coli* and purified as previously described (Zhao et al. 2015) (**Supplementary Fig. S3C, D**). Highly purified recombinant proteins of D53, SKP1−D3 and D14 were incubated with 50 μM *rac*-GR24 and then subjected to gel-filtration chromatography on a Hiload Superdex 200 16/600 GL column from GE Healthcare, followed by SDS−PAGE analysis. In the size-exclusion chromatography experiment, the proteins were eluted in three peaks. Peak 1 contained all components of the D53−SKP1−D3−D14 complex, while peak 2 and peak 3 corresponded to the SKP1−D3−D14 complex and D14, respectively (**Fig. 1B**). Fractions in peak 1 formed homogeneous particles with a diameter of ∼13 nm in the negative staining analysis (**Fig. 1C**). The particle feature of the D53−SKP1−D3−D14 complex was significantly improved compared to apo-D53. To evaluate the thermostability of the complexes, we performed dynamic light scattering (DLS) and compared the aggregation onset temperature (Tonset), which is a marked temperature point indicating protein denaturation and aggregation. Compared with apo-D53, the D53−SKP1−D3−D14 complex exhibited significantly enhanced thermostability as the Tonset value increased from 34°C to 43°C (**Fig. 1D**). The addition of either glutaraldehyde or glutaraldehyde together with NP40 could increase the thermostability of the D53−SKP1−D3−D14 complex, as evidenced by an increase in Tonset by ∼10°C, but cryo-EM detection of the glutaraldehyde crosslinked D53−SKP1−D3−D14 complex did not obtain a better resolution (**Fig. 1D**, **Supplementary Fig. S1G**). Together, we reconstituted the D53−SKP1−D3−D14 complex in vitro with higher thermostability than apo-D53 and observed homogeneous particles in negative staining.

### Structure of SKP1−D3−D14 in the presence of D53

**Fig. 2:**
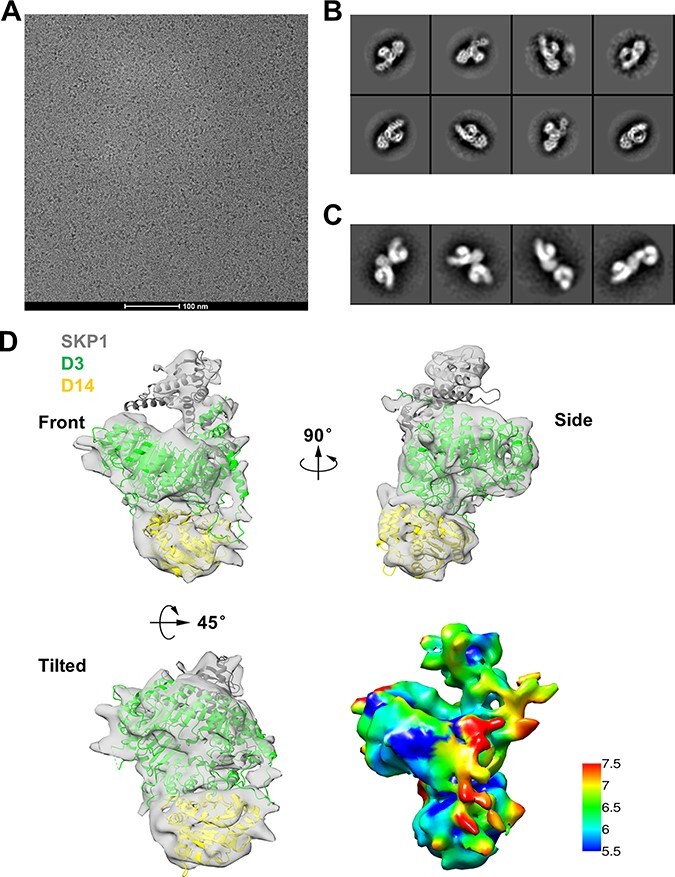
The overall EM structure of SKP1−D3−D14 in complex with D53. (A) A representative cryo-EM macrograph of the D53−SKP1−D3−D14 complex. The scale bar is 100 nm. (B) Representative views of 2D class averages of the SKP1−D3−D14 complex. (C) Representative views of 2D class averages of the SKP1−D3−D14 complex with extra density. (D) The overall structure of the SKP1−D3−D14 complex. Three different views are shown on the front, side and titled. SKP1 is colored in gray, D3 in green and D14 in yellow. The resolution range is color-coded.

Cryo-EM data collection and image processing were performed for the D53−SKP1−D3−D14 complex (**Fig. 2A**). Initially, 332,886 particles were extracted from 2,678 micrographs, and 93,466 individual particles were further used for reference-free two-dimensional (2D) classification (**Supplementary Fig. S4A**). Raw micrographs and 2D class averages showed that most particles displayed monomers (**Fig. 2B**). However, through reference-free 2D classification using a larger mask diameter, some 2D classifications showing a double-comma shape were enriched, and the captured extra density likely belonged to the D53 protein (**Fig. 2C**). Unfortunately, D53 was too flexible to capture in a certain form, and the electron density information was lost during average and classification processing. After 3D classification, a subset of 42,499 particles remained and was used for reconstruction, yielding a final cryo-EM map with a resolution of 7.09Å estimated by the gold Fourier shell correlation standard (**Supplementary Fig. S4A–C, Table S1**).

To further optimize the sample of complex D53−SKP1−D3−D14, we expressed and purified the D2 domain of D53 (residues 718–1,131) and reconstituted complex D53^D2^−SKP1−D3−D14 in vitro. His-D3 pull-down assays showed that 50 μM *rac*-GR24, GR24^4DO^ and GR24^5DS^ could induce the association among D3, D14 and D53^D2^ (**Supplementary Fig. S5A**). In the size-exclusion chromatography experiment, the first peak contained all components of D53^D2^−SKP1−D3−D14 and was subjected to negative staining analysis in the presence or absence of 0.01% v/v glutaraldehyde (**Supplementary Fig. S5B, C**). The particles of the D53^D2^−SKP1−D3−D14 complex showed compact and homogeneous features comparable to those of the D53−SKP1−D3−D14 complex (**Supplementary Fig. S5C, D**). The cryo-EM micrographs displayed homogeneous particles and 219 micrographs were collected, resulting in approximately 260,000 particles of the D53^D2^−SKP1−D3−D14 complex (**Supplementary Fig. S5E**). However, the 2D classification only displayed the density map of D3, and the information of neither D14 nor D53^D2^ was clarified (**Supplementary Fig. S5F**).

Based on the classification data of the D53−SKP1−D3−D14 complex and D53^D2^−SKP1−D3−D14 complex, we performed deep analysis on the cryo-EM data of the D53−SKP1−D3−D14 complex. The distribution of particle angle indicated an orientation preference, leading to the removal of approximately half of the particles (**Supplementary Fig. S4A, C**). The cryo-EM map showed relatively distinct secondary structural elements, and an atomic model of SKP1−D3−D14 was built using the predicted structure of SKP1−D3 and D14 from the Alphafold2 database (https://alphafold.com/) as references. D14, D3 and SKP1 had good fits to the refined map. Unfortunately, front, tilted and side views of the cryo-EM map did not reveal the location of D53 (**Fig. 2D**, **Supplementary Table S1**).

**Fig. 3:**
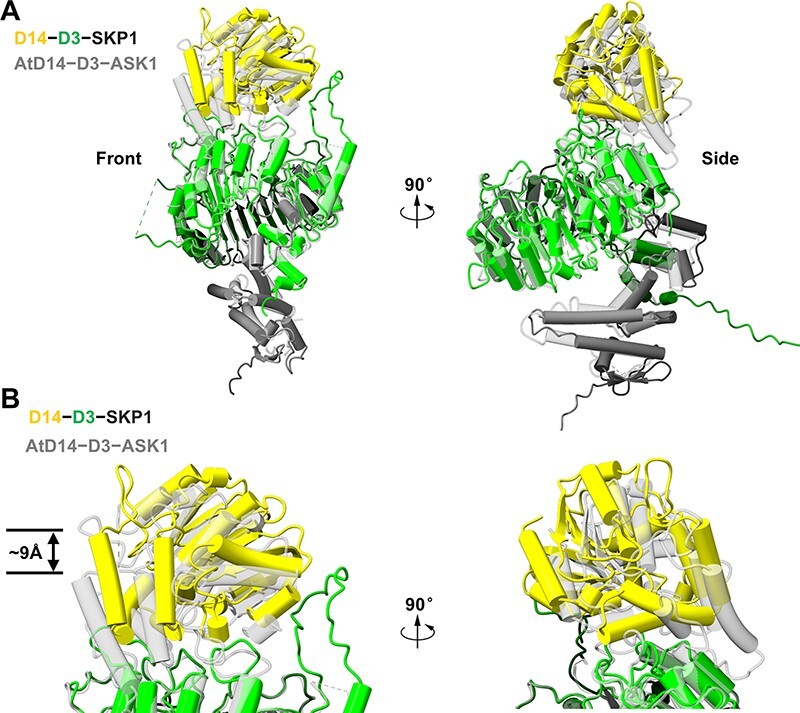
The structural comparison between the SKP1−D3−D14 and ASK1−D3−AtD14 complexes. (A) Superposition of D53-bound SKP1−D3−D14 and ASK1−D3−AtD14 (PDB code 5HZG) in front view and side view. (B) A close-up view of the interface between D14 and D3 in different complexes with their orientations related to (A). D14 and D3 in the D53-bound SKP1−D3−D14 complex are colored yellow and green, respectively. The ASK1−D3−AtD14 complex is colored gray.

The cryo-EM structure of the D53-bound complex SKP1−D3−D14 was superimposed on the crystal structure of ASK1−D3−AtD14 (Protein Data Bank, code 5HZG). No large conformational rearrangement was observed between the structures of the two complexes (**Fig. 3A**). However, the local conformations of D14−D3 and AtD14−D3 showed variation (**Fig. 3A, B**). Compared with the ASK1−D3−AtD14 complex, D14 in the SKP1−D3−D14 complex was flipped away from D3 at ∼9Å (**Fig. 3B**). Although the density does not have enough details to allow for accurate fitting of the SKP1−D3−D14 structure and the approximate 9Å rotation may relate to the difference in D14 proteins from rice and *Arabidopsis*, the local conformational differences are still possibly associated with the formation of holo-complex.

### Allosteric effects correlated with assembly of the D53−SKP1−D3−D14 complex

**Fig. 4:**
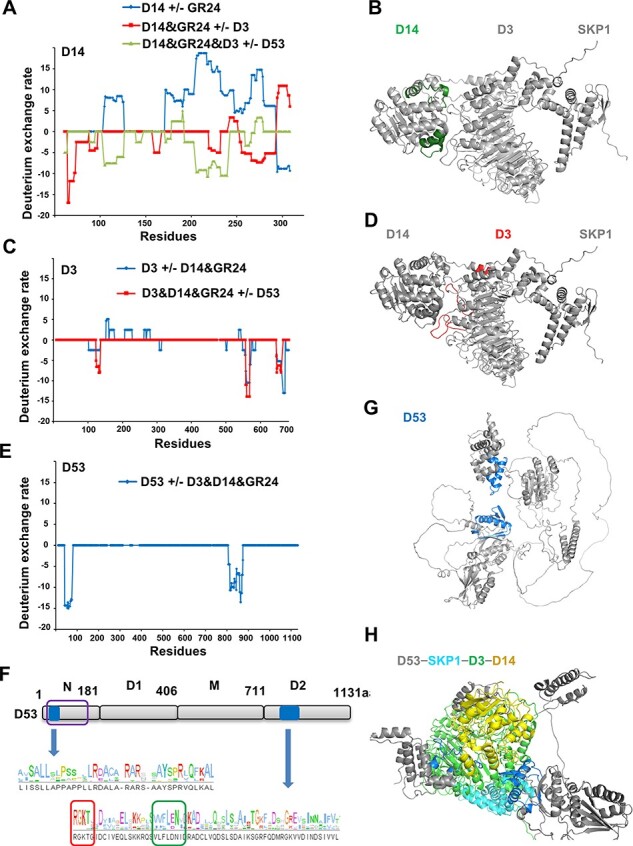
Allosteric effects correlated with the assembly of the D53−SKP1−D3−D14 complex. (A) Comparative HDX–MS studies of D14 upon 250 µM *rac*-GR24, D3 and D53 interactions. (B) Residues that are protected with the addition of D53 are mapped onto the D14 structure and colored green. (C) Comparative HDX–MS studies of D3 upon D14 and D53 interactions. (D) Residues that are protected by adding D53 are mapped onto the D3 structure and colored red. (E) Comparative HDX–MS studies of D53 upon D14−D3 interaction. (F) Two potential binding regions in D53 are colored blue and listed in detail. The ClpN, RGKT and walker B motifs are boxed in purple, red and green squares, respectively. (G) Residues that were protected after incubation with D3&D14&*rac*-GR24 are mapped onto the calculated D53 structure and colored blue. (H) Modeled structure of the D53−SKP1−D3−D14 complex. D14, D3 and SKP1 are colored yellow, green and cyan, respectively. D53 without long loops is colored gray and the two potential binding regions of D53 are colored blue. In (A), (C) and (E), a negative or positive value on the Y axis (D%) represents protection (stabilization) or deprotection (destabilization) against deuterium exchange in the corresponding region of the D14 (A), D3 (C) or D53 (E) protein depicted on the X axis when a binding event takes place. Differences in D% between −5% and 5% are considered nonsignificant. In (B), (D) and (G), residues without significant changes are colored gray.

To examine conformational transitions of D14, D3 and D53 in the assembling process induced by *rac*-GR24, we carried out HDX−MS analysis in which the secondary structure and buried elements within the complex exchange slower than exposed loops. A positive value in the rate of hydrogen to deuterium exchange (D%) represents destabilized conformational changes in the presence of new components, while a negative value represents protected conformational changes. We first examined the conformational changes of D14 incubated with *rac*-GR24, D3 and D53 (**Fig. 4A**, **Supplementary Fig. S6**). Compared with the state of apo-D14, addition of *rac*-GR24 induced destabilization of regions L103-A124 and I170-L292 coupling stabilization of region Q293-A308 in D14 (**Supplementary Fig. S6A**, **Fig. 4A**, blue line). When D3 was further added, the Q293-A308 region stabilized by *rac*-GR24 became loose, indicating that D3 allosterically disrupted the engagement of the Q293-A308 region. The neighboring motif A258-F291 was protected, which contains the catalytic triad residue D268 and the homologous region of AtD14 K216-A223 that showed disappeared electron density in the structure of ASK1−D3−AtD14 (Yao et al. 2016), indicating that A258-F291 may be involved in the formation of the D14−D3 complex (**Supplementary Fig. S6B**, **Fig. 4A**, red line). Furthermore, the influence of D53 on HDX activity of the A258-F291 region showed no statistical significance (**Supplementary Fig. S6C**, **Fig. 4A**, green line), suggesting that the conformation of at least specific regions of D14 related to the ASK1–D3–AtD14 complex is probably sustained during the binding of D53. In addition, the supply of D53 mainly induced the stabilization of regions L103-A124 and A204-F225 in D14 (**Supplementary Fig. S6C**, **Fig. 4A**, green line). These two regions consisted of four helixes near the D3−D14 interface and were mapped onto the protein structure of D14 (**Fig. 4B**, green regions).

To illuminate the dynamics of D3, we analyzed changes between D3 and the *rac-*GR24–induced D3−D14 complex. The regions of A593-S608 and L684-F709 in D3 were protected, which partially overlapped with D3^CTH^ (E693-D720) and included the interfaces between D3 and D14 in the crystal structure of ASK1−D3−AtD14 (**Supplementary Fig. S7A**, **Fig. 4C**, blue line). The addition of D53 further stabilized the D3 regions of Y123-F134, G595-D606 and L684-F698 (**Supplementary Fig. S7B**, **Fig. 4C**, red line), which were mapped onto the protein structure of D3 (**Fig. 4D**, red region). Among these stabilized regions, G595-D606 and L684-F698 are located near the SKP1−D3−D14 complex interface, and the L684-F698 region includes six amino acids of the CTH motif. This potential interface location is consistent with the absence of a major surface conformational change between the D53-bound complex SKP1−D3−D14 (**Fig. 2C**) and the ASK1−D3−AtD14 structure (PDB code 5HZG) although some residues of D3^CTH^ also participate in the conformational changes during the formation of the D3−D14 and D53−D3−D14 complexes.

Furthermore, we analyzed the conformational changes of D53 in the absence and presence of D3−D14 upon *rac*-GR24 treatment and identified two regions with increased stability (**Fig. 4E**, **Supplementary Fig. S8**). I41-D75 is located in the double Clp-N domain of D53, and K814-S875 contains the walker B motif and the RGKT motif, which are essential for ubiquitination and degradation of D53 (**Fig. 4F**) (Jiang et al. 2013, Zhou et al. 2013). The structure of D53 was predicted using Alphafold2 (Jumper et al. 2021), and the protected regions of D53 displayed clear secondary structures. The mapped region in D53^N^ domain consists of three helices and the mapped region in D53^D2^ domain contains two helices and four β sheets (**Fig. 4G**, blue region). Consistently, the D53^D2^ domain, which contains region K814-S875, can form a complex with D3 and D14 in vitro (**Supplementary Fig. S5A, B**), but the 2D classification of the cryo-EM map only captured the density map of D3 rather than the location of D14 or D53^D2^ domain (**Supplementary Fig. S5F**), suggesting that other regions such as the N domain of D53 may be important for stabilization of the signal transduction complex. Sequence alignment of D53, D53-like, SMXL6, SMXL7 and SMXL8 demonstrated that the potential binding regions in D53 are highly conserved (**Supplementary Fig. S9**).

Taken together, we detected protection on D14 residues (L103-A124 and A204-F225), D3 residues (Y123-F134, G595-D606 and L684-F698) and D53 residues (I41-D75 and K814-S875) in the formation of the D53−SKP1−D3−D14 complex and mapped these regions to the structures of D14, D3 and D53 (**Fig. 4A****–****G**, **Supplementary Figs. S6–8**). The protected regions of D14 and D3 mainly lie around the interfaces of D14 and D3 in the SKP1−D3−D14 complex, suggesting that D53 may also bind the interfaces of D14 and D3 in a manner dependent on the SL-induced D14−D3 assembly.

## Discussion

Full-length D53 contains many disordered structures (**Fig. 4G**), such as long loops between the inner domains and inter-domains, which may interfere with the calculation of D53**−**SKP1−D3−D14 complex. We found that the protected regions in the D53^N^ and D53^D2^ domains (D53^ND2^ for short) during the process of D53−SKP1−D3−D14 complex formation are highly conserved among homologs from 25 plant species (**Fig. 4F**, **Supplementary Fig. S10A, B**), suggesting that these regions may be involved in the direct interaction with SKP1−D3−D14. To investigate how D53 interacts with SKP1−D3−D14, we first calculated and predicted the structure of D53^ND2^-bound SKP1−D3−D14 complex using the cryo-EM structure of SKP1−D3−D14 and ND2 domains of D53 with the docking method HoDock (Gong et al. 2010). Based on the predicted complex structure, we took advantage of biochemical information to rearrange the positions of D53^ND2^ domains and minimize the complex global energy using Gromacs-2022 molecular dynamics package (https://doi.org/10.5281/zenodo.6451564). To obtain the holo-complex model, the relative positions of D53^ND2^ domains were used to calibrate the relative locations of full-length D53, which was then aligned to the D53^ND2^−SKP1−D3−D14-modeled complex. In the modeled structure of D53−SKP1−D3−D14 complex, the interaction between D53 and D3−D14 was stabilized by the D53^N^ and D53^D2^ domains from different directions (**Fig. 4H**). The potential binding regions of D14 and D3 were around the D3−D14 interface observed by HDX−MS, suggesting that D53 may interact with the SKP1−D3−D14 complex along their interface and slightly crowed out D14. The proposed model is consistent with the observation that compared with the ASK1−D3−AtD14 complex, D14 was flipped away from D3 in the D53-bound SKP1−D3−D14 complex. More genetic and structural biology analyses are required to evaluate the state of D14 in the formation of holo-complex and the recruitment of D53 for subsequent ubiquitylation and degradation. Interestingly, the architecture of residues L45–L74 in the D53^N^ domain experienced significant conformational transitions compared with apo-D53. Residues L45–L74 in apo-D53 consisted of three helices (**Fig. 4G**), while L45–L74 in the modeled D53−SKP1−D3−D14 complex allosterically stretched into a long loop, which is located around the catalytic triad amino acids and may influence the D14 hydrolysis activity (**Supplementary Fig. S10C**).

Previous studies have suggested that the D2 domain in D53 is necessary and sufficient for the degradation of rice D53 upon *rac*-GR24 treatment (Shabek et al. 2018). The deletion of the conserved RGKT motif or P-loop stabilized D53, SMXL6, SMXL7, SMAX1, SMXL2 and OsSMAX1 in vivo (Jiang et al. 2013, Zhou et al. 2013, Soundappan et al. 2015, Wang et al. 2015, 2020b, Liang et al. 2016, Khosla et al. 2020, Zheng et al. 2020), and loss of RGKT motif impaired the interaction of D53^D2^ domain with D3^CTH^ and D14 (Shabek et al. 2018). However, the full-length SMAX1 had a much shorter half-life than its D2 domain in *Arabidopsis* although SMAX1^D2^ retained the ability to be targeted and degraded by MAX2 (Khosla et al. 2020), suggesting that the stability of SMAX1 may also be regulated by unknown domains. In this study, we found that the stability of the D53^D2^−SKP1−D3−D14 complex was worse than that of the D53−SKP1−D3−D14 complex in the cryo-EM assay (**Supplementary Fig. S5F**, **Fig. 2B, C**). More importantly, HDX−MS analysis showed that both N-terminal and D2 domains of D53 contain amino acids with conformational changes, indicating that the D53^D2^ domain is necessary but insufficient to accomplish the complex assembly and SL signaling. The assembly of holo-complex may undergo a sandwich binding process, in which the interaction with D53^N^ domain pushed D14 away and D53^D2^ domain attached to the interface of SKP1−D3−D14 (**Fig. 4H**). Notably, the proposed model cannot rule out the involvement of other regions in the formation of the signaling complex. For instance, the D1M domain of SMAX1 in *Arabidopsis* was required for the direct interaction with the KAI2 Ligand (KL) receptor KAI2 and determined the interaction specificity of SMAX1 and SMXL7 with KAI2 and D14 in yeast (Khosla et al. 2020).

The conformational transition regions of D14 upon the binding of D53 showed overlapped regions with the D14−D3 interaction sites in the structure of ASK1−D3−AtD14 (Yao et al. 2016) and the amino acids of D3^CTH^ (Shabek et al. 2018). More importantly, D53 binding induced allosteric effects on undiscovered residues, such as N110-A124, A204-T207, Y209 and G215-F225 (**Fig. 4B**). The mutation of AtD14^P169L^, a conserved residue of D14^P219^, leads to deficiency in SL perception and dwarf phenotypes in *Arabidopsis thaliana* (Chevalier et al. 2014). These results suggested that the conformational changes in the process of D53 binding are important for SL signaling and plant development.

The interaction motifs identified in this study provide insight into engineering D53 proteins with minimized active motifs to mediate ubiquitination, degradation and SL signaling. Deep analyses of the conformational dynamics and structure of the D53−SKP1−D3−D14 complex will elucidate the elaborate mechanisms of SL perception in the near future.

## Materials and Methods

### Plasmid construction

Protein expression vectors were constructed or obtained as follows. To generate GST-D53^D2^ recombinant proteins, the coding sequences (CDS) of D53 (residues 718–1,131) was amplified using primers of D53-718-BamH1-5 and D53-E-Xho1-3T and cloned into PEGX-6P-1 vector. Rice D14 was a gift from E. Xu as previously described (Zhao et al. 2015). Constructs expressing the His-D53, His-D3 and SKP1 recombinant proteins were amplified using primers of D53-1-BamH1-5 and D53-E-Xho1-3T, primers of D3-1-BamH1-5 and D3-E-Sal1-3T and primers of SKP1-1-BamH1-5 and SKP1-E-Xho1-3T, respectively. Baculoviruses were generated using the pFastBac1 vector (Cat. No. 10360014, Invitrogen, Carlsbad, CA) based on published data (Liu et al. 2022). To co-express His-D3 and non-tagged SKP1 in insect cells, CDSs of SKP1 and His-D3 were amplified and digested with *Bam*HI and *Xho*I, and *Bam*HI and *Sal*I, and then cloned into the pFastBac1 vector using a Ligation High kit (Cat. No. LGK-201, Toyobo, Japan). The construct expressing D3 was amplified using primers of D3-1-BamH1-5 and D3-476-TEV-R and D3-514-TEV-F and D3-End-Sal1-3T with a procedure described previously (Shabek et al. 2018). Primers for constructs are listed in **Supplementary Table S2**.

### Bacmid DNA preparation, transfection and baculovirus packaging

The bacmid DNA preparation, transfection and baculovirus packaging of D53, D3 and SKP1 were conducted following a procedure described previously (Liu et al. 2022). Briefly, bacmid DNA expressing His-D53, His-D3 and SKP1 was prepared in *E. coli* DH10Bac-competent cells, transfected into Sf9 insect cells and cultured at 28°C for 96 h. The baculovirus package was produced and used to infect cultured insect cells.

### Protein preparation and purification

Full-length D53, full-length SKP1 and D3 lacking disordered loops (residues 476–514) were expressed as 6×His fusion proteins in Sf9 insect cells. Protein was isolated from the soluble cell lysate by Ni Sepharose resin (Cat. No. 17-5318-01, GE Healthcare, Uppsala, Sweden). To remove the long linker, the SKP1–D3 complex was digested at 4°C by TEV protease (Cat. No. T4455, Sigma, St. Louis, MO) for 8 h and then purified by anion exchange and size-exclusion chromatography, yielding a split form of the SKP1–D3^NTD/CTD^ complex with SKP1, D3^NTD^ (residues 1–476) and D3^CTD^ (residues 514–720) based on published data (Shabek et al. 2018). Plasmids encoding His-D14 and GST-D53^D2^ were expressed in *E. coli* BL21 cells and purified using ion-exchange and size-exclusion chromatography as previously described (Zhao et al. 2015, Liu et al. 2022).

### DLS

DLS samples were prepared at 0.5–1.0 mg ml^−1^ and equilibrated for 5 min, and then, 10 μl of samples were loaded onto the Dyna Pro Nano Star (Wyatt Technology https://www.wyatt.com) as previously described (Duan et al. 2020). The intensity was recorded in a temperature range from 25°C to 70°C to evaluate the thermostability. Data analyses are supported by the Dynamics software Dyna Pro Nano Star.

### TSA

The thermostability of D53 was evaluated by the TSA as previously described (Niesen et al. 2007). In brief, 1,000 × SYPRO Orange (Cat. No. 200-664-3, Sigma-Aldrich, St. Louis, MO) was mixed with D53, and thermal melting curves were detected with a thermal ramp from 25°C to 100°C using a LightCycler 480 II Real-Time PCR System (Roche Diagnostics, Rotkreuz, Switzerland), and the ramp rate was set to 1°C.

### Negative-stain electron microscopy screening

The uranyl formate solution and glow-discharged grids were prepared as previously described (Duan et al. 2020). In brief, 10 µl of purified D53 and its related complexes were applied to the grids and washed twice. Negative-stain image screening and data collection were operated at 120 kV on Tecnai G2 Spirit transmission electron microscope (FEI, Thermo, Waltham, MA) at a nominal magnification of 10,500 with the defocus values ranged from −0.5 to −2.5 µm.

### Cryo-EM sample preparation and data collection

For cryo-EM analysis, 4 μl of D53 protein and D53−SKP1−D3−D14 complex at a concentration of 0.5–1 mg ml^−1^ were applied to glow-discharged (HARRICK PLASMA, Ithaca, NY) holey carbon grids (Quantifoil Cu 1.2/1.3, 400 mesh) and then blotted and flash-frozen by FEI Vitrobot Marked IV. Cryo-EM data were collected in super-resolution mode with the defocus values varied from −0.7 to −0.9 μm following the methods in a previous report (Wang et al. 2019).

### Image processing and 3D reconstruction

The raw super-solution dose-fractionated images were analyzed as previously described (Wang et al. 2019). Approximately 2,000 manually picked sets of particles were used to calculate 2D classification and further generate templates for reference-based particle picking. We summarized the parameters for data processing and 3D reconstruction in **Supplementary Fig. S4 and Table S1**.

### Model building and refinement

The separated atomic structure models of D3 and D14 were predicted with Alphafold2 and then docked in the cryo-EM map in ChimeraX (https://www.cgl.ucsf.edu/chimerax/). The merged model was refined in real space using the PHENIX software package (https://phenix-online.org/). We provided model statistics in **Supplementary Table S1**.

### HDX–MS analysis and data rendering

Peptides were identified by liquid chromatography tandem mass spectrometry (LC–MS/MS) with Thermo Fisher Orbitrap Fusion Mass Spectrometer. The raw data were analyzed by Proteome Discover 2.4 software to identify high-confidence peptides. For further HDX–MS analysis, 5 µM purified D14, 250 µM *rac-*GR24, 5 µM purified D3 and 5 µM purified D53 were added sequentially as indicated and then incubated for 30 min in buffer containing 50 mM HEPES and 50 mM NaCl (pH 7.5). Four microliters of protein/protein complex with ligand/peptide was diluted into 16 µl exchange buffer containing 50 mM HEPES (pH 7.5) and 50 mM NaCl in D_2_O, incubated for various HDX time points (e.g. 0, 10, 60, 300 and 900 s) at 4°C and then quenched by mixing with 20 µl of ice-cold 3 M GuHCl with 1% trifluoroacetic acid. The quenched samples were subjected to HDX–MS analysis following the procedure in a previous study (Song et al. 2021). The percentage of deuterium incorporation and statistical significance (unpaired *t-*test) were calculated and rendered by HDX Workbench software (http://hdxworkbench.com/) (Pascal et al. 2012). The HDX data from all overlapping peptides were consolidated to individual amino acid values using a residue-averaging approach (Song et al. 2021).

### Pull-down of recombinant proteins

In His-D3 pull-down assays, 0.5–1 mg of His-D3, D14 and D53^D2^ were incubated with 2 µM GR24^5DS^, GR24^4DO^ or *rac*-GR24 for 20 min at 4°C with gentle rotation. Then, 30 µl Ni-NAT agarose (Thermo Fisher Scientific, Waltham, MA) was added to the mixtures and incubated for 20 min. The agarose beads were washed 5 column volumes with cold lysis buffer (20 mM HEPES pH 7.5 and 150 mM NaCl), and proteins were eluted by elution buffer supplemented with 250 mM imidazole (Cat. No. 56748, Sigma) for 5 min. Input, wash and elution samples were analyzed by SDS−PAGE.

## Supplementary Material

pcad067_Supp

## Data Availability

The data underlying this article are available in [Electron Microscopy Data Bank] and can be accessed with [EMD-35402].
