# Supplementary material for: Conformational Dynamics of the D53−D3−D14 Complex in Strigolactone Signaling
Source: Plant Cell Physiol. 2023 Jun 29;64(9):1046–56. doi: 10.1093/pcp/pcad067 (PMC10858650; doi:10.1093/pcp/pcad067)
Supplement: pcad067_Supp [file pcad067_supp.zip › suppl_data/pcp-2023-e-00040-File006.pdf]

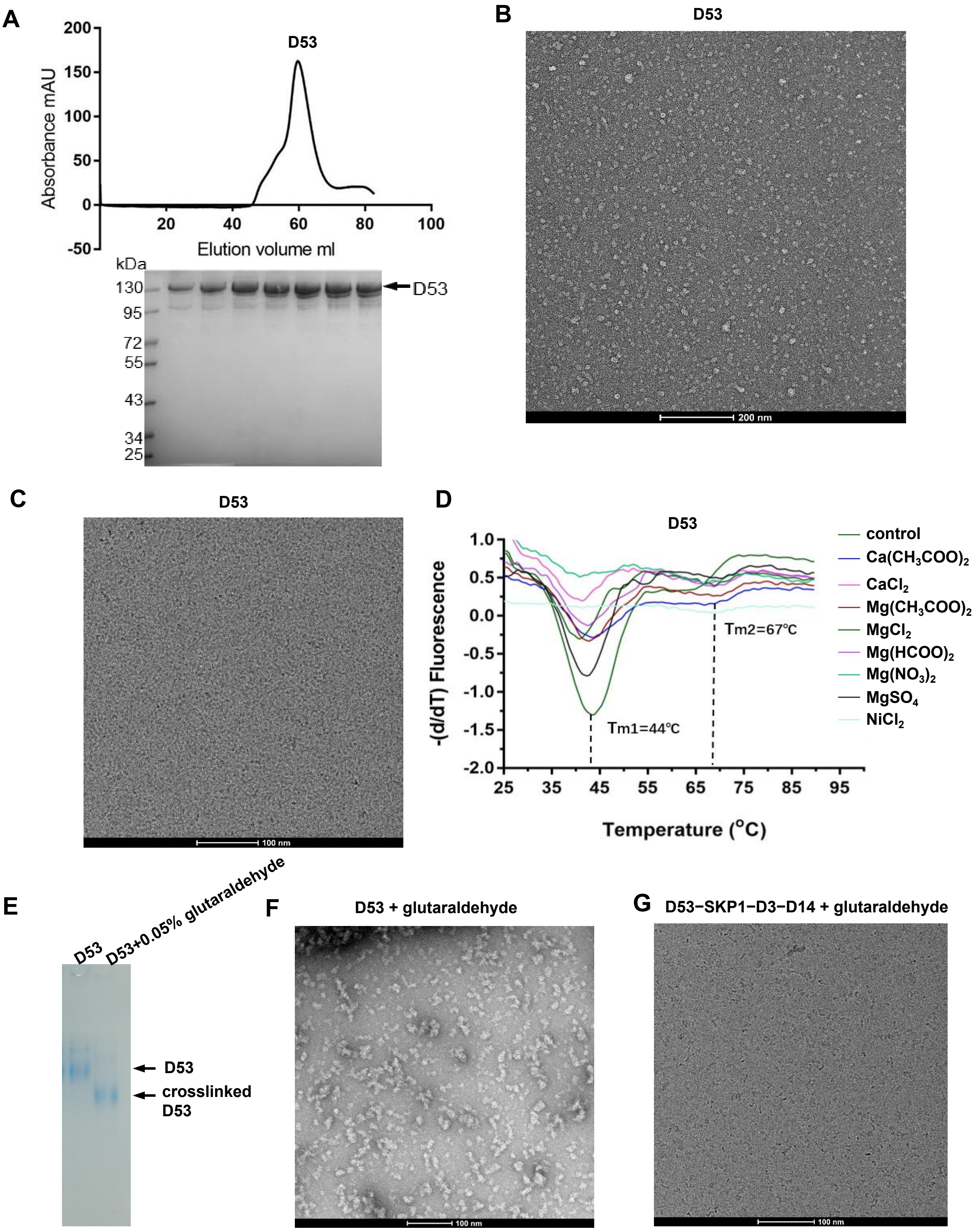

Supplementary Fig. S1 Characterization of D53 and its complex.
(A) Size-exclusion chromatography elution profile of the D53 protein. The peak indicates the elution position resolved in SDS–PAGE followed by Coomassie blue staining. The calculated mass of D53 monomer is 123 kDa. (B) Representative negative staining image of D53. The scale bar is 200 nm. (C) Representative cryo-EM micrograph of D53. The scale bar is 100 nm. (D) Thermal shift assays (TSA) of full length D53. The thermal stabilities of standard buffer condition (20 mM HEPES, pH 7.0, 100 mM NaCl, 5% (v/v) glycerol) and incubation with various bivalent cations were evaluated by TSA. Color-coded as labeled using the method described in the methods section. (E) Native gel analysis of protein D53 and D53 incubated with 0.05% (v/v) glutaraldehyde. (F) Representative negative staining image of D53 with 0.05% (v/v) glutaraldehyde. The scale bar is 100 nm. (G) Representative cryo-EM micrograph of the D53−SKP1−D3−D14 complex with 0.05% (v/v) glutaraldehyde. The scale bar is 100 nm.

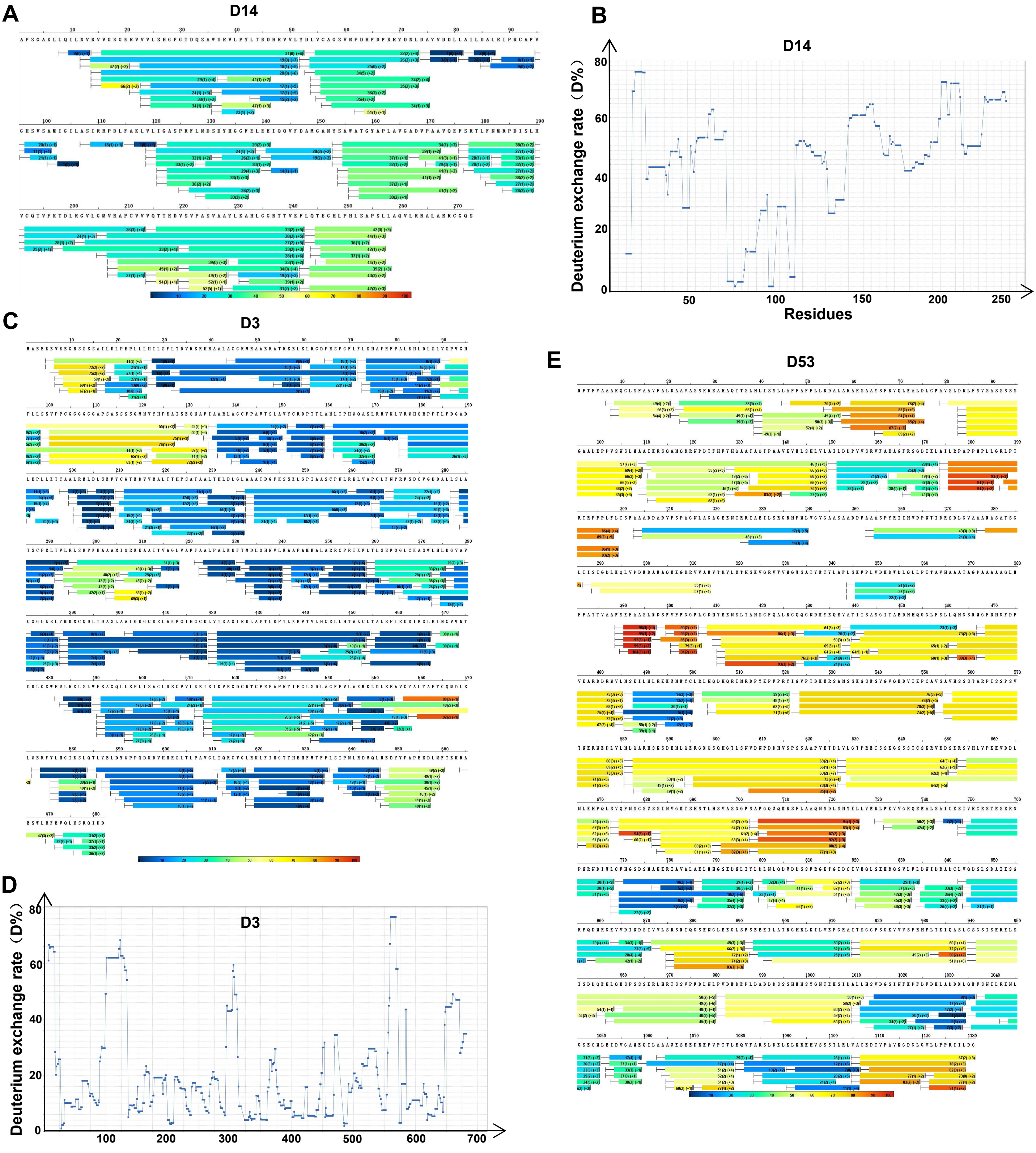

Supplementary Fig. S2 HDX dynamics of apo-D14, D3 and D53.

(A) HDX deuterium exchange heatmap of apo-D14. The bars below the sequence represent the peptide fragments resolved by mass spectrometry (91.9% coverage). (B) Plot view of apo-D14 hydrogen deuterium exchange. (C) HDX deuterium exchange heatmap of apo-D3. The bars below the sequence represent the peptide fragments resolved by mass spectrometry (97.4% coverage). (D) Plot view of apo-D3 hydrogen deuterium exchange. (E) HDX deuterium exchange heatmap of apo-D53. The bars below the sequence represent the peptide fragments resolved by mass spectrometry (92.4% coverage). In (A), (C), (E), the color of each peptide according to the smooth color gradient HDX perturbation key (D%) is shown in each indicated figure. Regions that show high HDX activity are colored orange and red; regions that show low HDX activity are colored green and blue.

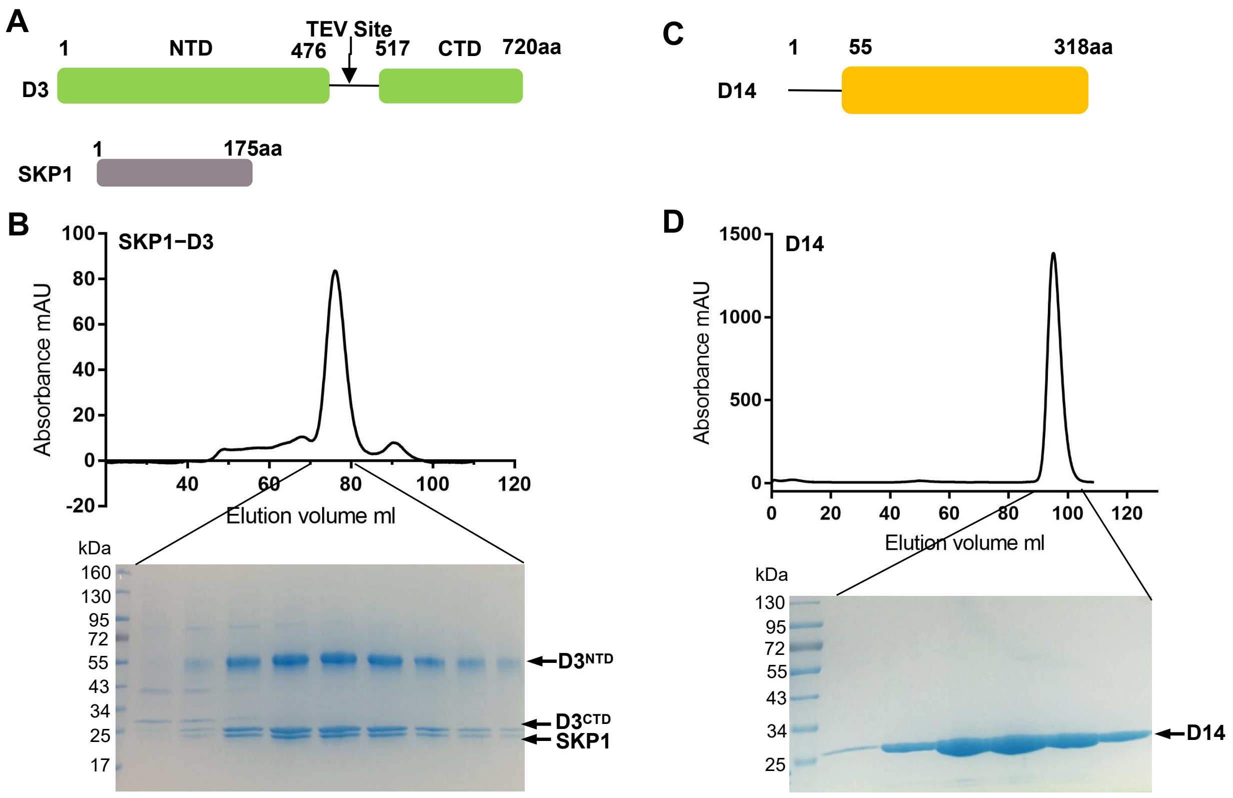

Supplementary Fig. S3 Purification of the SKP1−D3 complex and D14.

(A) Schematic representation of rice D3 and SKP1 constructs used in this study. $D3^{NTD}$ and $D3^{CTD}$ represent the N-terminal domain and C-terminal domain of D3 respectively. (B) Size-exclusion chromatography elution profile of SKP1−D3. The peak indicates the elution position resolved by SDS–PAGE followed by Coomassie blue staining. The calculated mass of a SKP1−D3 monomer is 85 kDa. (C) Schematic representation of the rice D14 construct used in this study. (D) Size-exclusion chromatography elution profile of D14 (residues 55-318). The peak indicates the elution position resolved by SDS–PAGE followed by Coomassie blue staining. The calculated mass of a D14 monomer is 29 kDa.

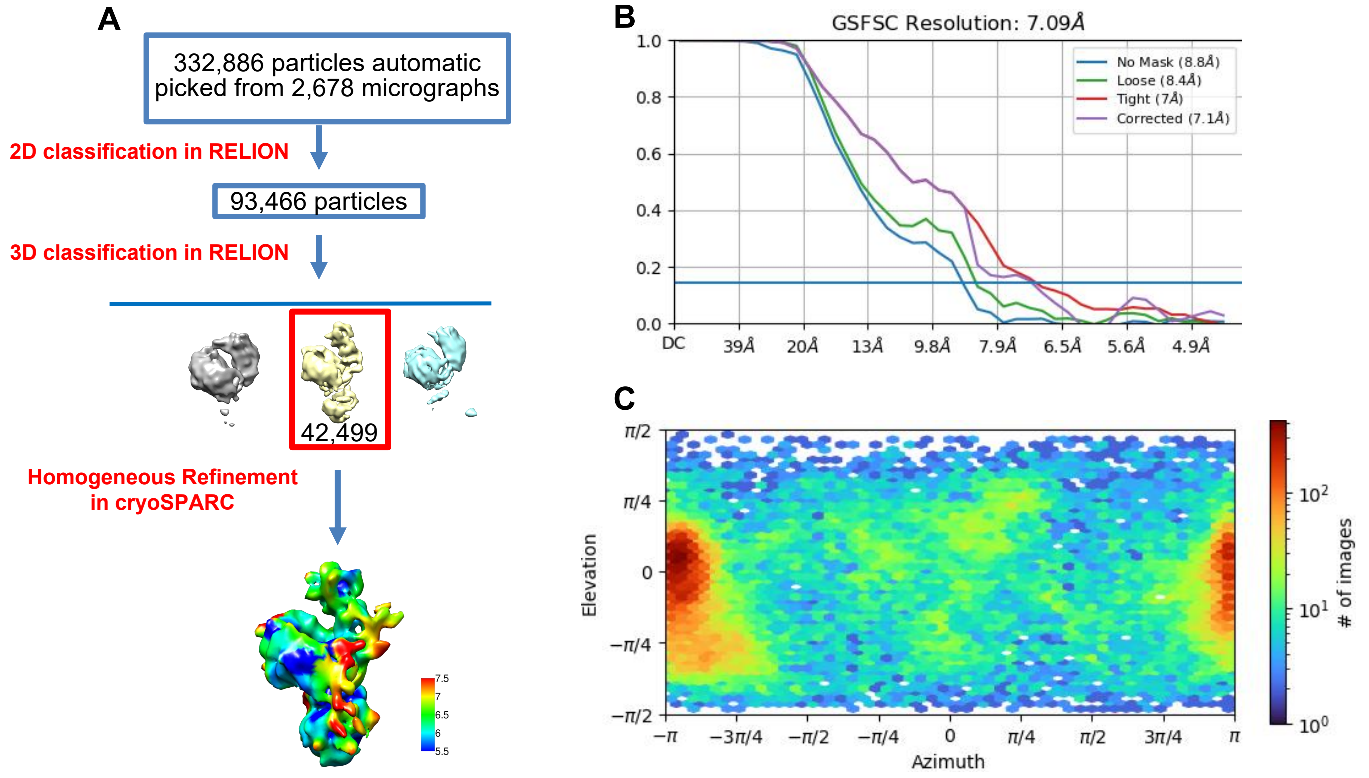

Supplementary Fig. S4 3D reconstruction of the SKP1−D3−D14 complex.

(A) Flowchart for cryo-EM data processing and 3D reconstruction of the SKP1−D3−D14 complex. (B) FSC curves at 0.5 and 0.143 of the 3D reconstruction of the SKP1−D3−D14 complex. (C) Particle angle distribution for the final 3D auto-refine.

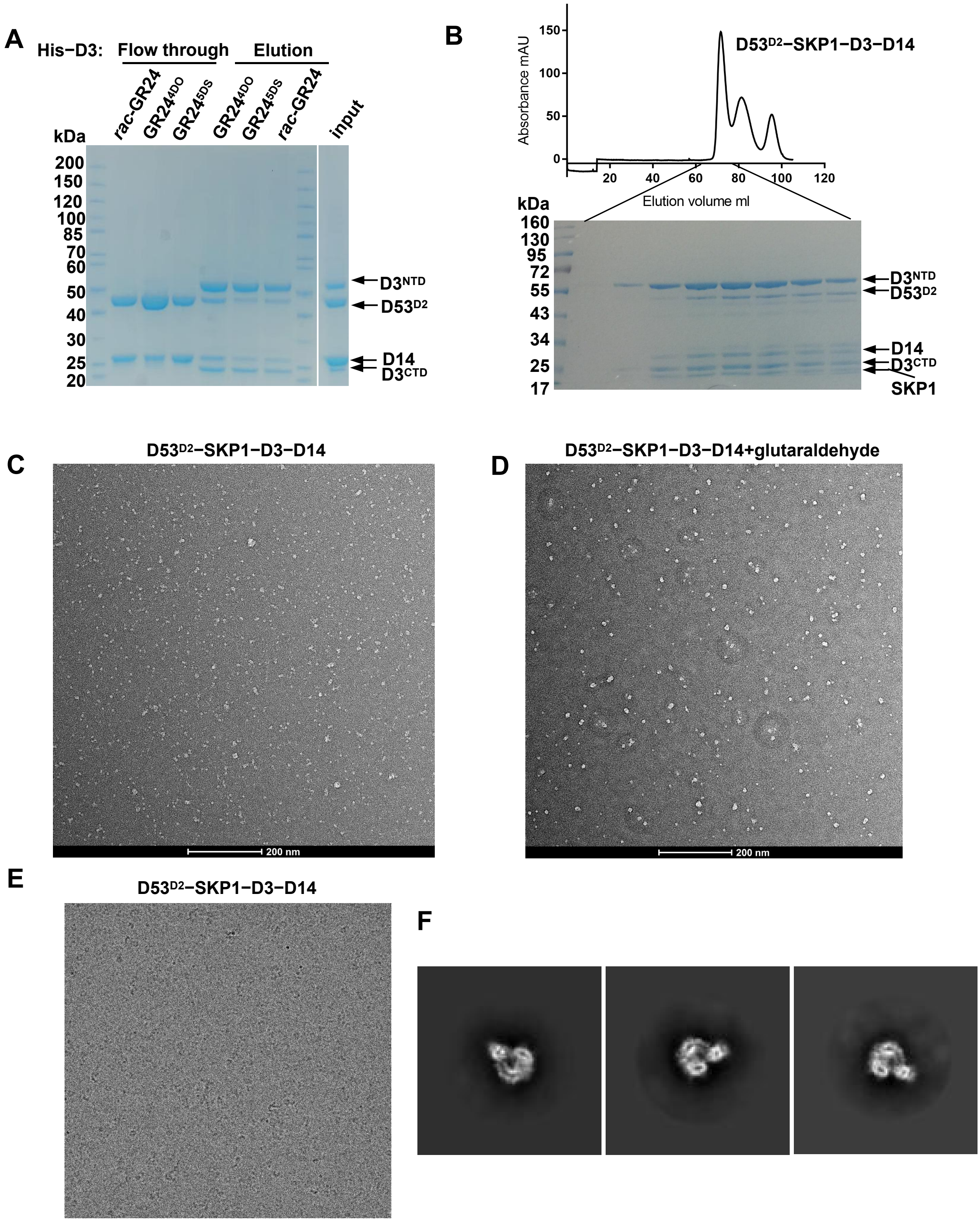

Supplementary Fig. S5 Interactions among D53^{D2}, SKP1−D3 and D14.

(A) His-D3 pull-down assay using highly purified recombinant His-D3, untagged D53^{D2}, untagged D14 and analogs of SLs. (B) Size-exclusion chromatography analysis of the interaction between the D2 domain of D53 and SKP1−D3−D14 complex. (C) Representative negative staining image of the D53^{D2}−SKP1−D3−D14 complex. The scale bar is 200 nm. (D) Representative negative staining image of D53^{D2}−SKP1−D3−D14 complex with 0.01% (v/v) glutaraldehyde. The scale bar is 200 nm. (E) Representative cryo-EM micrograph of the D53^{D2}−SKP1−D3−D14 complex. (F) 2D classification of complex D53^{D2}−SKP1−D3−D14.

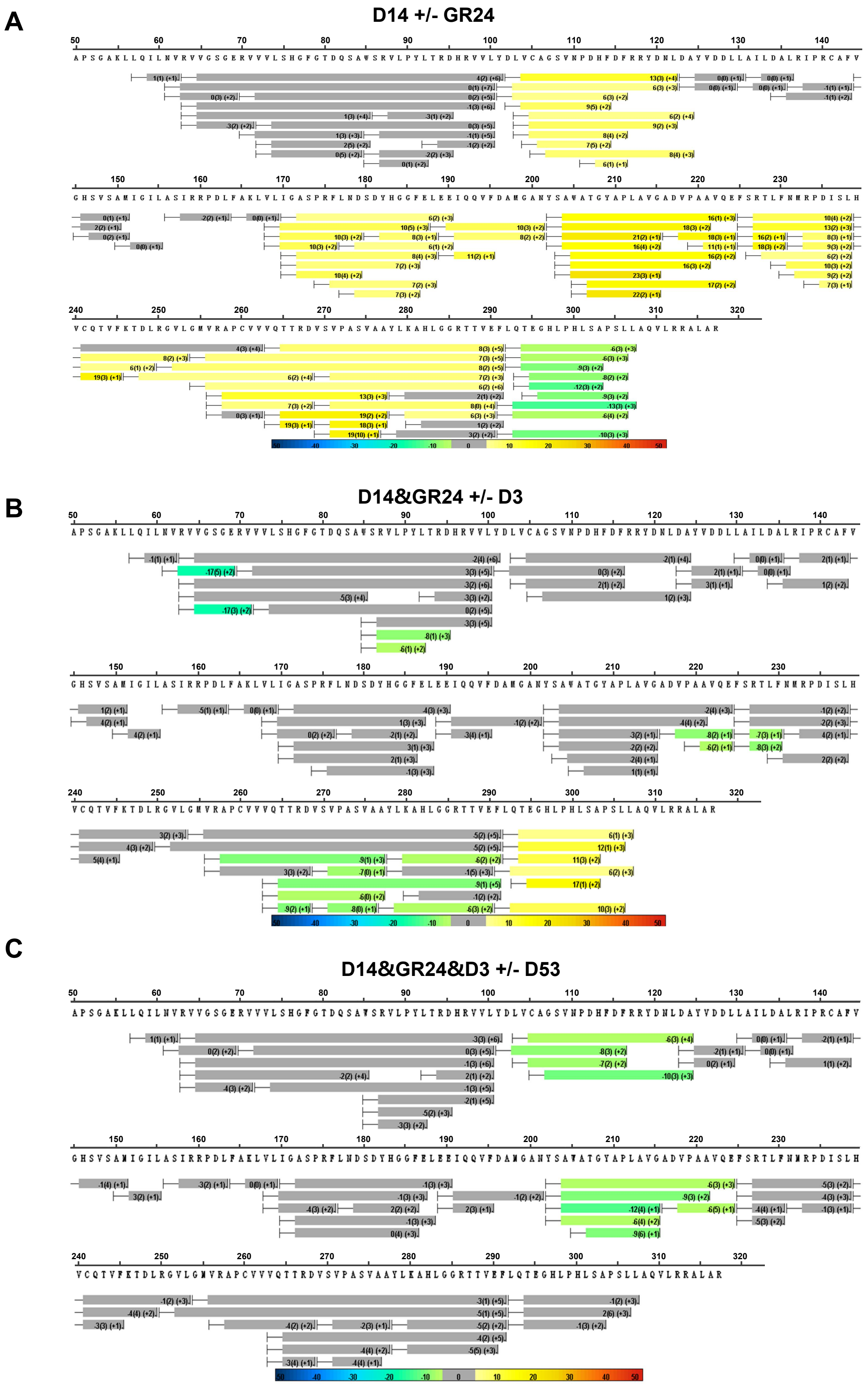

Supplementary Fig. S6 Dynamics analysis of D14 in the D53−SKP1−D3−D14 complex.
(A) Hydrogen-deuterium exchange (HDX) perturbation heatmap comparison between apo-D14 and D14 upon *rac*-GR24. (B) HDX perturbation heatmap comparison between D14&*rac*-GR24 and D14&*rac*-GR24 incubated with D3. (C) HDX perturbation heatmap comparison between D14&*rac*-GR24&D3 and D14&*rac*-GR24&D3 incubated with D53. The bars below the sequence represent the peptide fragments resolved by mass spectrometry and the color of each peptide according to the smooth color gradient HDX perturbation key (D%) is shown in each indicated figure. Regions that show elevated HDX activity are colored yellow; regions that show decreased HDX activity are colored green; regions that show no statistically significant changes between compared states are colored gray. Rice D14 protein with residues 55–318 was expressed, purified and used for HDX analysis.

A

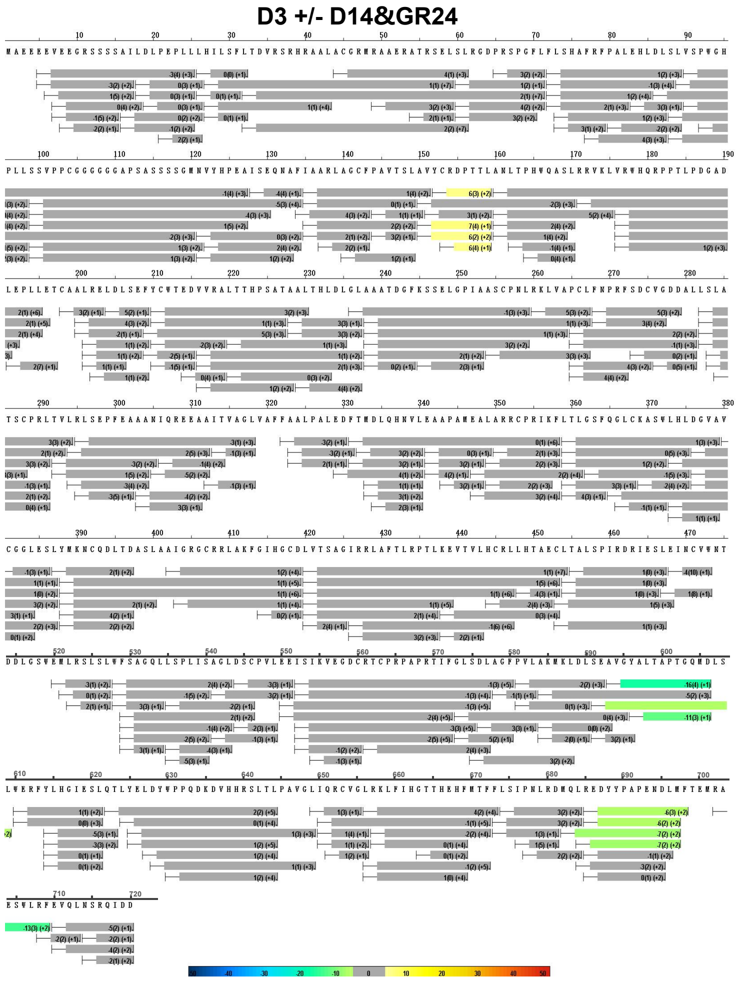

B

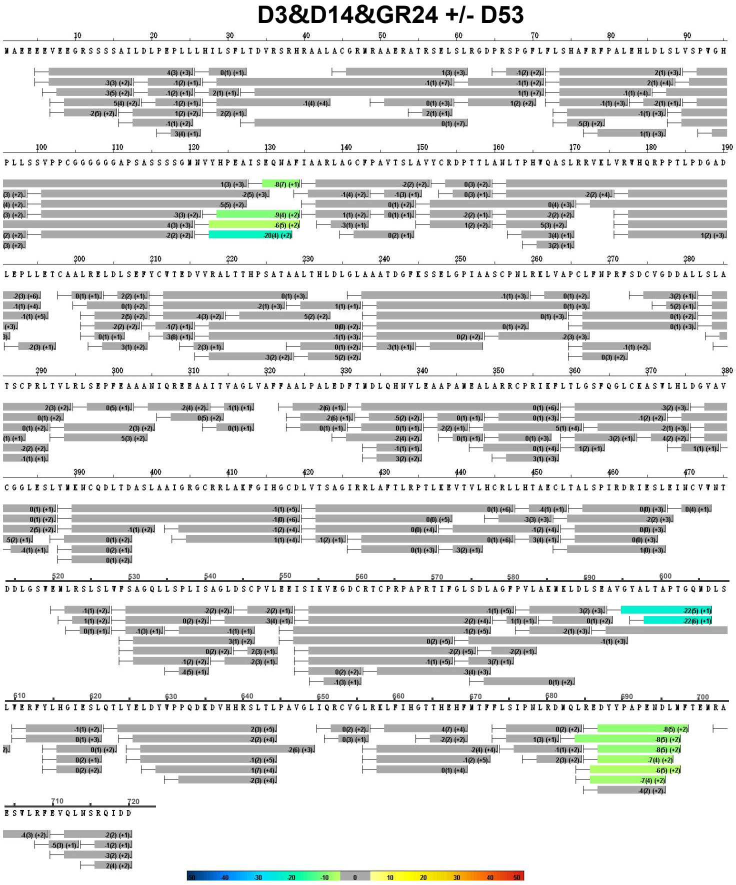

Supplementary Fig. S7 Dynamics analysis of D3 in the D53−SKP1−D3−D14 complex.

(A) HDX perturbation heatmap comparison between apo-D3 and D3 incubated with D14&*rac*-GR24. (B) HDX perturbation heatmap comparison between D3&D14&*rac*-GR24 and D3&D14&*rac*-GR24 incubated with D53. The bars below the sequence represent the peptide fragments resolved by mass spectrometry and the color of each peptide according to the smooth color gradient HDX perturbation key (D%) is shown in each indicated figure. Regions that show elevated HDX activity are colored yellow; regions that show decreased HDX activity are colored green and blue; regions that show no statistically significant changes between compared states are colored gray. Rice D3 protein lack of residues 477-516 was expressed, purified and used for HDX analysis.

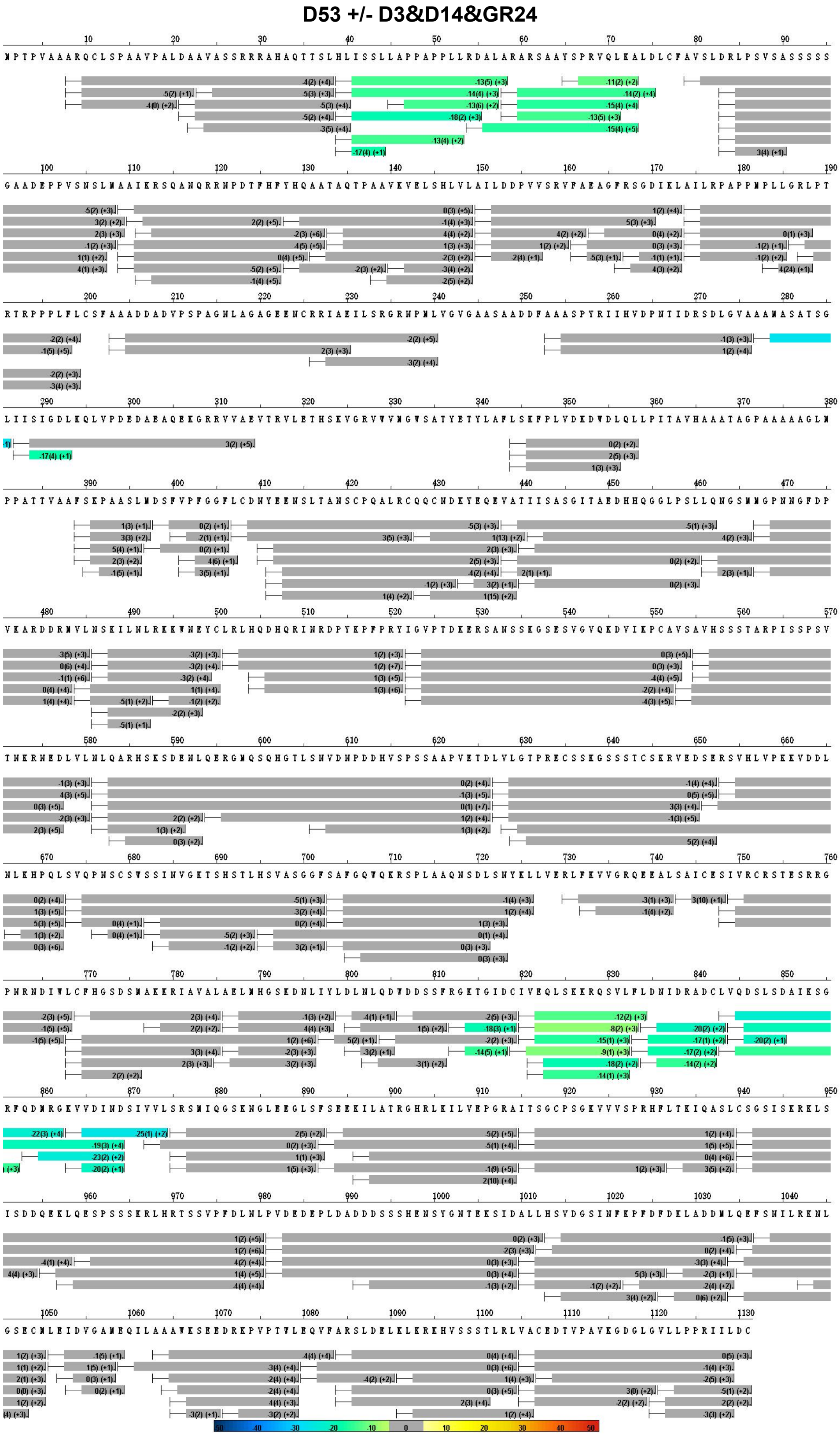

Supplementary Fig. S8 Dynamics analysis of D53 in the D53−SKP1−D3−D14 complex.
HDX perturbation heatmap comparison between apo-D53 and D53 incubated with D3&D14&*rac*-GR24. The bars below the sequence represent the peptide fragments resolved by mass spectrometry and the color of each peptide according to the smooth color gradient HDX perturbation key (D%) is shown in indicated figure. Regions that show decreased HDX activity are colored green and blue; regions that show no statistically significant changes between compared states are colored gray. Full-length D53 protein was expressed, purified and used for HDX analysis.

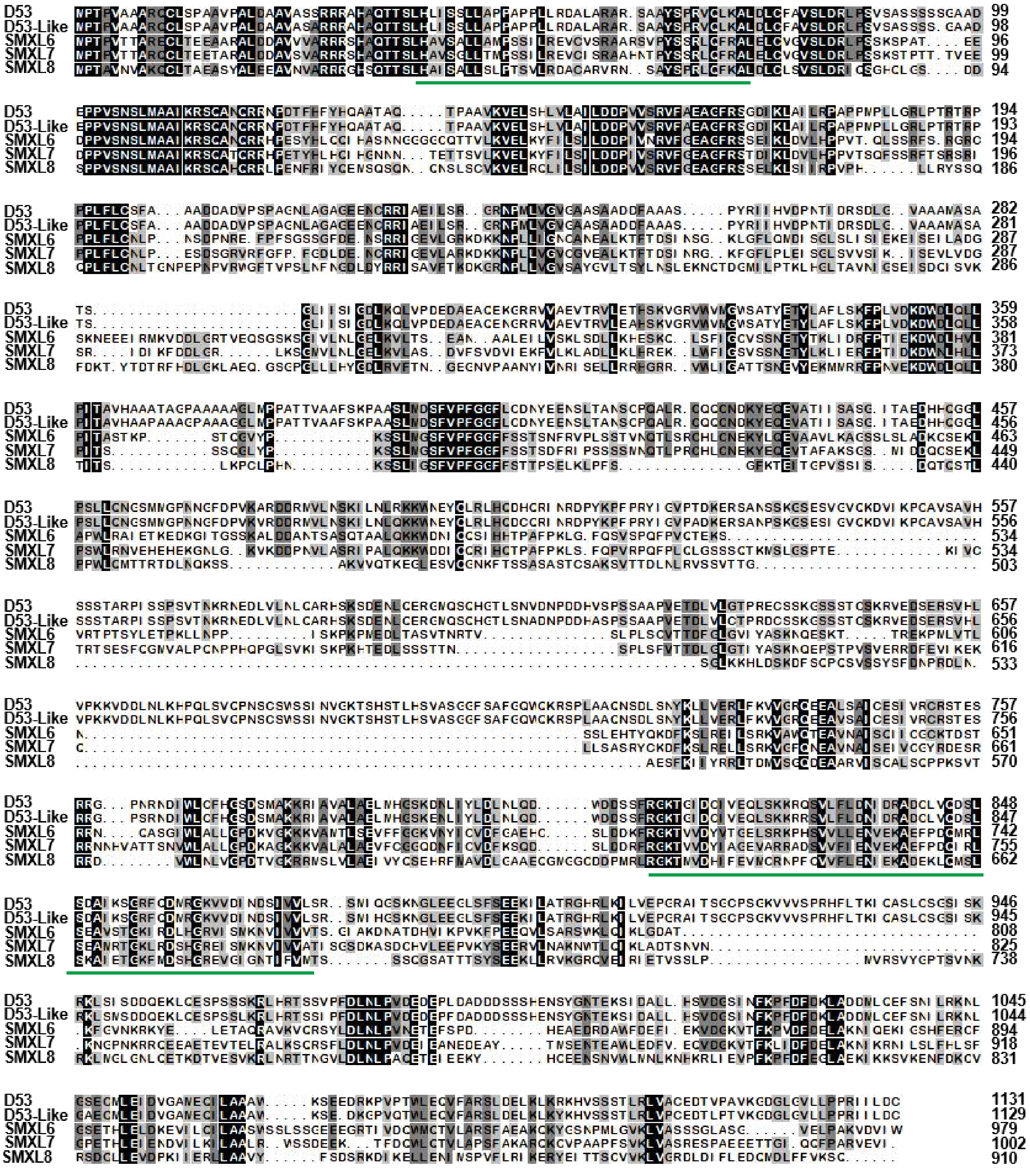

Supplementary Fig. S9 Multiple sequence alignment of D53 orthologues among different species.
Identical and conserved residues are highlighted by black and gray grounds, respectively. GenBank accession numbers for sequences from top to bottom: D53 (Q2RBP2) and D53-like (Q2QYW5) in *Oryza sativa*, SMXL6 (Q9LML2), SMXL7 (O80875) and SMXL8 (F4IGZ2) in *Arabidopsis thaliana*. The conformational transition motifs in D53 identified by HDX-MS in the process of D53−SKP1−D3−D14 complex formation are indicated by solid green lines.

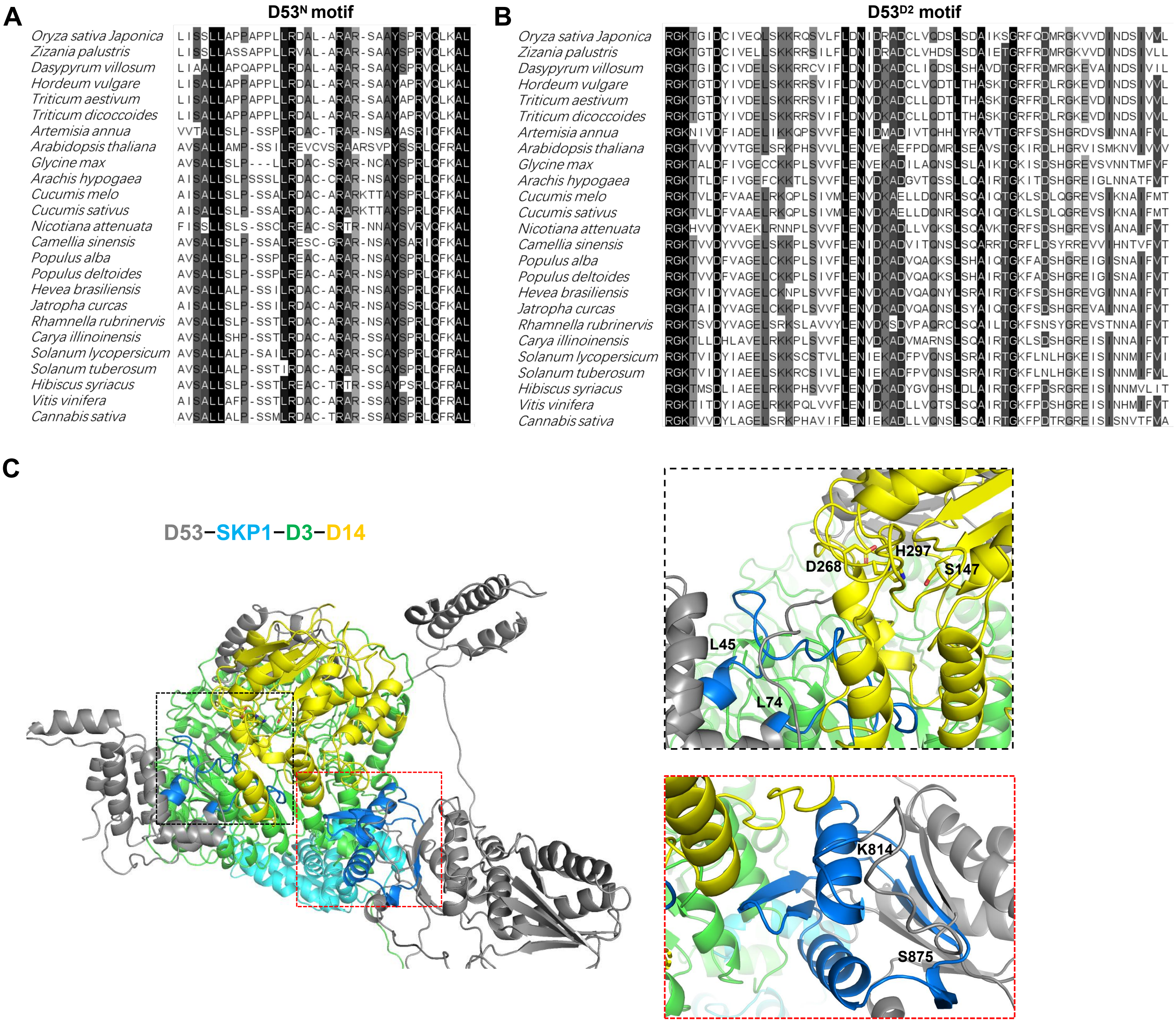

Supplementary Fig. S10 D53 potential binding motifs and the modelled complex interfaces.
(A and B) Sequence alignment of interacting regions in D53^{N} domain (A) and D53^{D2} domain (B) among 25 orthologues from different plant species. The GenBank accession numbers for protein sequences from top to bottom are *Oryza sativa* (Q2RBP2), *Zizania palustris* (KAG8050869.1), *Dasypyrum villosum* (QIB02021.1), *Hordeum vulgare* (KAE8809081.1), *Triticum aestivum* (XP_044374360.1), *Triticum dicoccoides* (XP_037421411.1), *Artemisia annua* (PWA55782.1), *Arabidopsis thaliana* (Q9LML2), *Glycine max* (XP_040867945.1), *Arachis hypogaea* (XP_025681977.1), *Cucumis melo* (XP_008441470.1), *Cucumis sativus* (XP_011656414.1), *Nicotiana attenuata* (XP_019239544.1), *Camellia sinensis* (XP_028086466.1), *Populus alba* (XP_034923755.1), *Populus deltoides* (KAH8501390.1), *Hevea brasiliensis* (XP_021655572.1), *Jatropha curcas* (XP_012087333.1), *Rhamnella rubrinervis* (KAF3433416.1), *Carya illinoinensis* (KAG2730521.1), *Solanum lycopersicum* (XP_004247009.1), *Solanum tuberosum* (KAH0636097.1), *Hibiscus syriacus* (XP_039017358.1), *Vitis vinifera* (XP_002279036.1) and *Cannabis sativa* (KAF4369223.1). Identical and conserved residues are highlighted by black and gray grounds, respectively. (C) Modelled D53−SKP1−D3−D14 complex. D14 is colored yellow, D3 is colored green, D53 is colored gray except for its potential binding motifs, which is colored blue. Close-up views of the D53^{N} and D53^{D2} binding motifs are boxed in black and red dashed lines, respectively. Catalytic triad amino acids of D14 in the modelled complex are labeled.
