# Supplementary material for: Conformational Dynamics of the D53−D3−D14 Complex in Strigolactone Signaling
Source: Plant Cell Physiol. 2023 Jun 29;64(9):1046–56. doi: 10.1093/pcp/pcad067 (PMC10858650; doi:10.1093/pcp/pcad067)
Supplement: pcad067_Supp [file pcad067_supp.zip › suppl_data/pcp-2023-e-00040-File007.pdf]

Supplementary Table 1. Cryo-EM statistics and model refinement

PDB ID	8IF6
EMDB ID	EMD-35402
**Data collection and processing**	
Microscope	Titan Krios
Detector	Gatan K2 with GIF Quantum (20eV slit)
CS (mm)	0.01
Magnification	64K
Pixel size (Å)	1.091
Electron dost (e^-/ Å^2)	50(32 frames)
Defocus range (µm)	-0.7 ~ -0.9
Phase shift (°)	80-130
Micrograph Number	2,678
**Reconstruction**	
Particles picked	332,886
Software	RELION, CryoSPARC
Particles refinement	42,499
Symmetry	C1
Resolution (Å)	7.09
Sharpening B-factor (Å^2)	141.6
**Refinement**	
Software	PHENIX
Model composition	
Number of atoms	8,690
Protein residues	1126
Nucleotides	0
Ligand	0
Bonds RMSD	
Bonds lengths (Å)	0.004
Bonds angles (°)	0.747
**Validation**	
MolProbity score	2.17
Clash score	23.82
Rotamer outliers (%)	0.00
C-beta outliers (%)	0.00
Ramachandran plot	
Favored (%)	95.62
Allowed (%)	4.29
Outlier (%)	0.09
Model vs. Data	
CC mask/box	0.58/0.85

Supplementary Table 2. Primers used in this study.

Primer Name	Sequence (5’-3’)
His-D53	
D53-1-BamH1-5	GGAATTGGATCC ATGCCCACTCCGGTGGCC
D53-A1899G-3	CGAACTACACTCCCGAGGGGTGCC
D53-A1899G-5	GGCACCCCTCGGGAGTGTAGTTCG
D53-E-Xho1-3T	GGAATTCTCGAGTCA ACAATCTAGAATTATTCTTGGCGG
His-D3	
D3-1-BamH1-5	GGAATTGGATCCATGGCGGAAGAGGAGGAGGT
D3-End-Sal1-3T	GGAATTGTCGACATCATCAATTTGCCGGCTG
D3-514-TEV-F	GAGAATCTGTACTTCCAGGGTATCTTGGAAGCTGGGAGAT
D3-476-TEV-R	ACCCTGGAAGTACAGATTCTCGGTTGTGTTCCAGACACAGT
SKP1	
SKP1-1-BamH1-5	GGAATTGGATCCATGGCGGCCGAGGCGGA
SKP1-E-Xho1-3T	GGAATTCTCGAGTCATTCGAAGGCCCACTGG
His-D14	
D14-55-BamH1-5	GGAATTGGATCCATGCTGCTGCAGATCCTGAAC
D14-318-Xhol1-3T	GGAATTCTCGAGTCAGTCGGGCGAGAGCGCGGC
GST-D53 ^{D2}	
D53-718-BamH1-5	GGAATTGGATCCATGAATTCTGATCTGAGCAATTAC
D53-E-Xho1-3T	GGAATTCTCGAGTCA ACAATCTAGAATTATTCTTGGCGG
